# Giant splenic cyst complicated by infection due to *Salmonella enterica* serovar Livingstone in a previously healthy adolescent male: a case report

**DOI:** 10.1186/s12879-022-07529-6

**Published:** 2022-06-18

**Authors:** Junyan Qu, Zhiyong Zong

**Affiliations:** 1 Center of Infectious Disease, West China Hospital (Huaxi), Sichuan University, Guoxuexiang 37, Chengdu, 610041 China; 2 Center for Pathogen Research, West China Hospital, Sichuan University, Chengdu, China

**Keywords:** *Salmonella* Livingstone, Splenic cyst, Splenic abscess, Case report

## Abstract

**Background:**

Splenic cyst complicated by non-typhoid *Salmonella* infection is rare in healthy individuals in the era of antibiotics. *Salmonella enterica* subsp. *enterica* serovar Livingstone causing infection of giant splenic cyst has not been previously reported.

**Case presentation:**

We report a case of giant splenic cyst (maximum diameter, 21 cm) complicated with *Salmonella* Livingstone infection, which resulted in splenic abscess, in a 16-year-old previously healthy adolescent male. The splenic abscess was successfully treated with ultrasonography-guided percutaneous drainage and antimicrobial therapy.

**Conclusion:**

Infection of splenic cyst may be caused by *S.* Livingstone in immunocompetent individuals. This case may help clinicians to raise awareness towards splenic abscess and highlights the importance of drainage and antimicrobial agents to avoid splenectomy.

**Supplementary Information:**

The online version contains supplementary material available at 10.1186/s12879-022-07529-6.

## Background

Splenic cysts are rare lesions with an incidence of 0.07% [1]. By etiology, splenic cysts are classified as parasitic and non-parasitic. The latter is further divided into primary/congenital, degenerative, neoplastic and traumatic [2]. Splenic cysts are usually asymptomatic and are found occasionally on imaging examination [3]. When cysts are large in size, symptoms of compressing adjacent structures may occur, and complications such as infection, rupture and/or haemorrhage could develop occasionally [4]. Splenic cysts can form splenic abscesses due to bacterial infection. The common pathogens of splenic abscess are streptococci, staphylococci, *Escherichia coli* and *Mycobacterium* spp. [5, 6]. Splenic abscess caused by *Salmonella enterica* subsp. *enterica* serovar Typhi, which causes typhoid fever, is uncommon with an incidence of 0.29–2%, while that caused by Non-typhoid *Salmonella* (NTS) is rare [7]. Only few cases of splenic cysts with NTS infection have been reported in literature [8, 9].

Salmonellosis due to *Salmonella enterica* subsp. *enterica* serotype Livingstone (*S.* Livingstone) is rare in human and is mainly recovered from poultry and poultry products [9]. In recent years, *S.* Livingstone has been increasingly isolated in some production settings and countries [10] and has caused outbreaks in some countries [11, 12]. In China, there was only one report about gastroenteritis due to *S.* Livingstone [13]. Splenic cysts complicated by *S.* Livingstone infection have not been previously reported. Here, we report a case of giant splenic cyst infected by *S.* Livingstone in a previously healthy adolescent.

## Case presentation

**Fig. 1:**
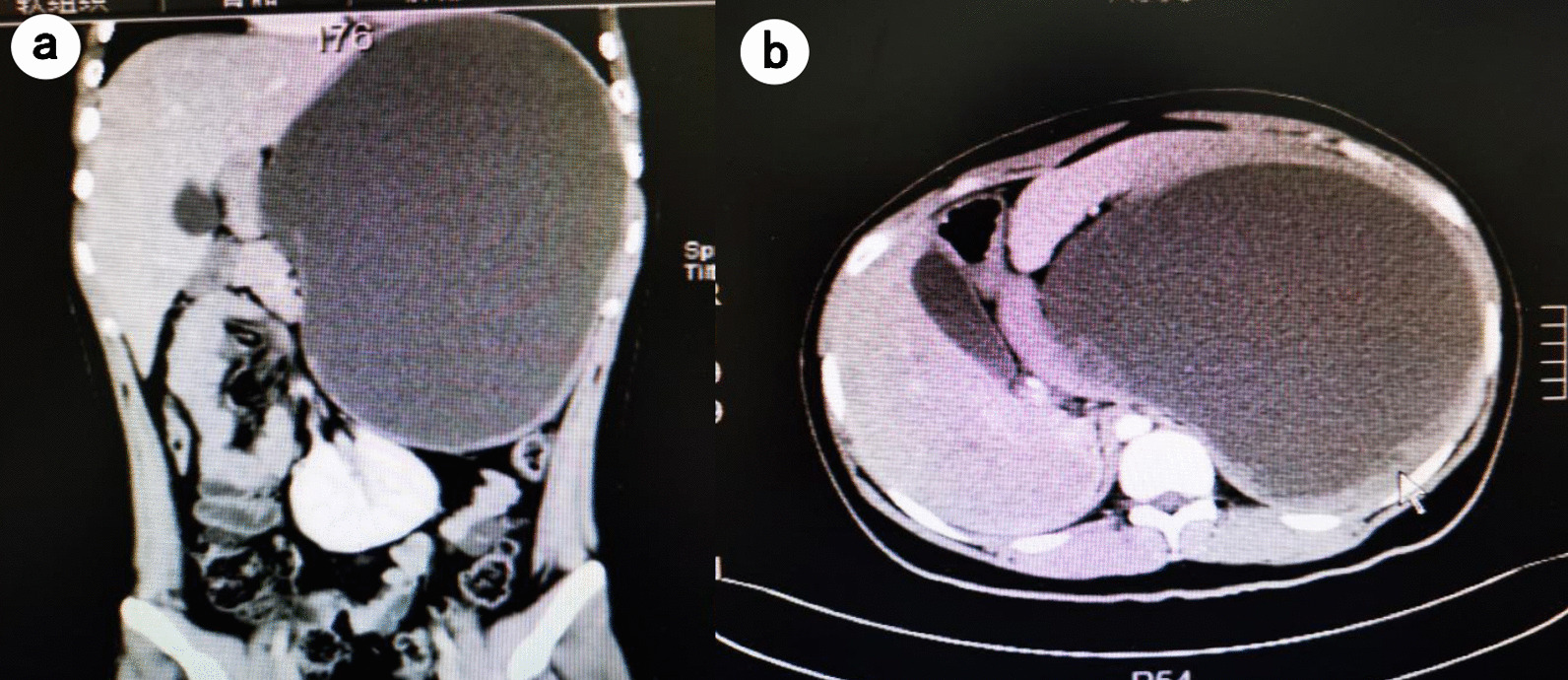
Abdominal CT taken at the local hospital 6 days prior to admission showed a 21 × 16 cm giant abscess in spleen

A 16-year-old male presented to the emergency department with abdominal pain and fever. He had no history of trauma nor exposure to echinococcosis. Twenty-two months ago, he had an abdominal ultrasound at a local hospital for health check, which revealed an 18.5 × 17.2 cm cystic mass in spleen. He had no symptoms and chose to neglect it. Six days ago, he was admitted to a local hospital due to sudden abdominal pain and high fever (39 °C) but without gastrointestinal symptoms. Abdominal computer tomography (CT) showed a 21 × 16 cm giant abscess in spleen (Fig. 1), and then a percutaneous drainage was performed. Purulent and bloody drainage was drained from the splenic abscess. After the drainage, he suffered from chest tightness and shortness of breath with decreased oxygen saturation and was transferred to our hospital.

On physical examination on admission, he looked acutely ill and his vital signs were: body temperature, 36.5 °C; heart rate, 94 beats/min; and respiratory rate, 24 breaths/min. Breath sounds in the lower left lung decreased. The right abdominal tenderness and rebound pain with muscle tension were noticed. Blood routine tests revealed elevated white blood cell (WBC) count of 26.08 × 10^9^/L (neutrophils, 93.9%) and platelet count of 358 × 10^9^/L and slightly lower hemoglobin of 129 g/L (normal range, 130–175 g/L). Blood inflammatory makers were also elevated with 0.58 ng/mL procalcitonin (PCT; normal range, < 0.046 ng/mL), 58 mm/h erythrocyte sedimentation rate (normal range, < 21 mm/h), and 61.70 mg/L C-reactive protein (CRP) level (normal range, < 5 mg/L). Blood chemistry showed a slightly decreased serum albumin level of 37.8 g/L (normal range, 40.0–55.0 g/L) and increased total bilirubin of 85.3 μmol/L (normal range, 5.0–28.0 μmol/L) and direct bilirubin of 65 μmol/L (normal range, < 8.8 μmol/L) but with normal alanine and aspartate aminotransferases. Results of serum tumor markers were 154 U/mL CA-199 (normal range, < 30 U/mL), < 1.5 U/mL CA-724 (normal range, < 6.5 U/mL), 1.73 ng/mL CEA (normal range, < 5 ng/mL), and 2.25 ng/mL AFP (normal range, < 7 ng/mL). Serum tests for human immunodeficiency virus (HIV) was negative. Serum antibodies against parasites including *Echinococcus* spp., *Taenia solium*, and *Schistosoma* spp. were negative. The absolute count and the proportion of T-lymphocyte subsets were also normal. Aerobic and anaerobic bacterial cultures of stool and blood were performed on admission but turned to be negative.

**Fig. 2:**
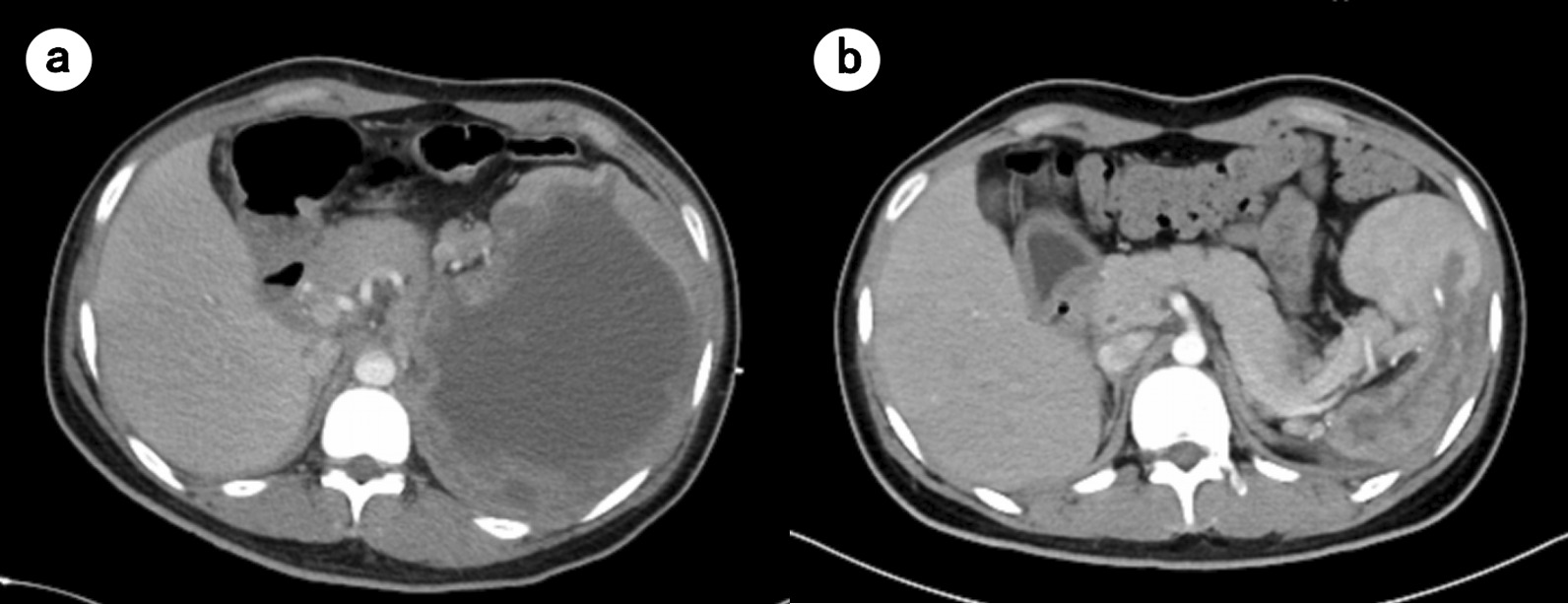
Abdominal CT scan on admission showed giant cystic lesion within the spleen (**a**). A follow-up abdominal CT taken 42 days later exhibited that the abscess had significantly decreased (**b**)

Imipenem (500 mg every 8 h) were empirically given. A CT scan of the chest revealed consolidation in both lower lungs (Additional file 1: Fig. S1), while an abdominal CT showed a splenic abscess with a drainage tube and signs of peritonitis (Fig. 2a). Examinations of the drainage fluid from the spleen mass indicated 30 × 10^6^/L WBC, 16,700 × 10^6^/L red blood cells, decreased glucose level of 0.02 mmol/L, and increased lactic dehydrogenase of 4254 IU/L. The drainage fluid of the splenic abscess was cultured, from which a bacterial strain was recovered without other microorganisms. Preliminary species identification by Vitek II (bioMérieux, Marcy-l'Étoile, France) assigned the strain to the genus *Salmonella*. The strain was subjected to genome sequencing using a HiSeq X10 platform (Illumina, San Diego, CA, USA). Antigenic profiling, multi-locus sequence typing, identification of antimicrobial resistance genes, and plasmid replicon typing were performed using genome analysis tools (SeqSero-1.2, MLST 2.0, ResFinder 4.1, and PlasmidFinder 2.1) available at Center for Genomic Epidemiology (http://genomicepidemiology.org/). Genome-based serotyping assigned the strain to *S.* Livingstone with an antigenic profile of 7:d:l,w (O antigen, O-7; H1 antigen, d; H2 antigen, l,w). Its draft genome sequence has been deposited in GenBank under accession no. JAGHKU000000000. The strain belongs to sequence type 543 (allele profile, *aroC*-*dnaN*-*hemD*-*hisD*-*purE*-*sucA*-*thrA*, 117-135-18-12-162-162-38), carries a single antimicrobial resistance gene, *aac(6′)-Iaa*, mediating resistance to aminoglycosides, and has a single known plasmid replicon Col(pHAD28)_1_KU674895. Virulence factors of the strain was predicted by querying the Virulence Factors of Pathogenic Bacteria (VFDB, http://www.mgc.ac.cn/cgi-bin/VFs/v5/main.cgi). The strain carried multiple virulence factors including fimbrial adherence determinants *csg*, *bcf*, *fim*, *saf*, *stb*, *stc*, *std*, *ste*, *stf*, *sth*, *sti* and *stk*, macrophage inducible gene *mig-14*, magnesium transport genes *mgtB* and *mgtC*, nonfimbrial adherence determinants *misL*, *ratB*, *shdA* and *sinH*, genes for SP1- and SP2-encoding type III secretion systems (*hil*, *iac*, *inv*, *org*, *prg*, *sic*, *sip*, *sop*, *spa*, *spi*, *sprB*, *spv*, *ssa*, *ssc*, *sse*, *ssr*), and afimbrial adhesin-encoding *afaB* and *afaC* (Additional file 2: Table S1). The strain was susceptible to third-generation cephalosporins, carbapenems and fluoroquinolones as determined using Vitek II (bioMérieux). Splenic abscess due to splenic cyst with *S.* Livingstone infection was diagnosed.

He received imipenem (500 mg every 8 h) for 10 days and then cefperazone-sulbactam (3 g every 8 h) for 30 days. About 500 mL bloody pus was drained from the splenic abscess daily. One month later, the spleen drainage tube was removed since no drainage fluid had been observed for 1 week. The abscess had significantly decreased according to the abdominal CT taken 42 days later (Fig. 2b). The patient recovered well and had no recurrent symptoms during the follow-up.

## Discussion and conclusions

Salmonellosis due to *S.* Livingstone in human is uncommon and giant splenic cysts complicated with *S.* Livingstone infection have not been previously reported. The most common clinical presentation of *S.* Livingstone infection was gastroenteritis [11]. *S.* Livingstone did not cause bacteremia due to the absence of the *spv* genes but could invade the adjacent organs from the intestine [14]. For splenic abscess, the most common clinical manifestations are fever, abdominal pain, left upper abdominal tenderness and splenomegaly [15], as seen in this case. Splenic cysts may act as a predisposing factor for splenic abscess. Many previous studies have shown that congenital epidermoid and mesothelial cysts are associated with increased CA-199 [1]. We did not have histopathological confirmation of splenic epithelioid cyst in this case. However, in light of his age, history, and significantly elevated serum CA-199, the most likely diagnosis was splenic abscess due to epidermoid cyst with *S.* Livingstone infection.

Like most NTS, *S.* Livingstone causes diseases mainly in individuals with underlying factors such as the immunocompromised status [16]. Patients with HIV infection, malnutrition, malaria, or inherited immunodeficiencies including chronic granulomatous diseases, sickle-cell diseases and B-cell deficiencies are at increased risks for invasive NTS (iNTS) diseases such as bacteremia and meningitis [17–19]. Host susceptibility to iNTS diseases varies significantly among individuals, and currently-available studies have focused on primary immunodeficiencies. The innate and acquired immune systems help the host to control of invasive *Salmonella* infection [20–23]. In addition, genetic variations may lead to susceptibility to different *Salmonella* species [20]. Although our patient did not have apparent immune deficiency, it cannot be excluded that he had genetic factors predisposing to the NTS infection.

Splenectomy was previously considered the gold standard for the treatment of splenic abscess [24]. However, recent studies have shown that percutaneous drainage combined with antimicrobial agents could be a better treatment option. Therapeutic splenectomy was required in only about 20% of patients [25, 26]. Splenectomy may lead to presumptive immune deficiency and increase the risk of bacterial infections and therefore should be avoided, especially in children. Percutaneous image-guided splenic intervention has also been found safer than splenectomy [27]. Splenic abscess could be single or multiple, with diameters ranging from 1 to 23 cm [28, 29]. Percutaneous aspiration and drainage have usually been performed in patients with a splenic abscess less than 10 cm in diameter [25, 30], while percutaneous drainage was successfully treated in this case with a spleen abscess of 21 cm in diameter. The most common complication of splenic intervention is hemorrhage [27]. The purulent and bloody drainage was initially drained from the splenic abscess of this case, which may reflect hemorrhage. Nonetheless, his drainage fluid became purulent and he had no bloody fluid after 10 days of drainage. Therefore, large unruptured splenic abscesses may be treated with percutaneous aspiration and drainage, while salvage splenectomy could be considered if other treatments fail. As for splenic cysts, different management approaches can be selected according to the clinical situation of the patient in the future, including percutaneous drainage, partial splenectomy, splenectomy, total cystectomy, laparoscopic decapsulation, marsupialization or cyst unroofing [4, 31]. Preserving splenunculus therapy, rather than splenectomy, may be preferred due to the immune function of the spleen.

In conclusion, splenic cyst can be complicated with *S.* Livingstone infection, resulting in splenic abscess, even in previously healthy adolescents. Percutaneous drainage and appropriate antimicrobial therapy can be used to treat large splenic abscess. This case of a previously healthy adolescent may help clinicians to raise awareness of splenic abscess and highlights the importance of drainage and antimicrobial agents to avoid splenectomy.

## Supplementary Information

**Additional file 1: Figure S1.** Chest CT on admission showed consolidation of lower lobes of both lungs (Panel a). Chest CT was normal after 20 days of antibacterial therapy (Panel b).

**Additional file 2: Table S1.** Virulence factors of the strain.

## Data Availability

Draft genome sequence of the strain has been deposited in GenBank under accession no. JAGHKU000000000. All other data are available in the text.

## Abbreviations

AFP: Alpha fetoprotein
CA: Carbohydrate antigen
CEA: Carcinoma embryonic antigen
CRP: C-reactive protein
CT: Tomography
HIV: Human immunodeficiency virus
iNTS: Invasive non-typhoidal Salmonella
NTS: Non-typhoid Salmonella
PCT: Procalcitonin
WBC: White blood cell
