# Supplementary figures and images for: Giant splenic cyst complicated by infection due to Salmonella enterica serovar Livingstone in a previously healthy adolescent male: a case report
Source: BMC Infect Dis. 2022 Jun 18;22:557. doi: 10.1186/s12879-022-07529-6 (PMC9206239; doi:10.1186/s12879-022-07529-6)

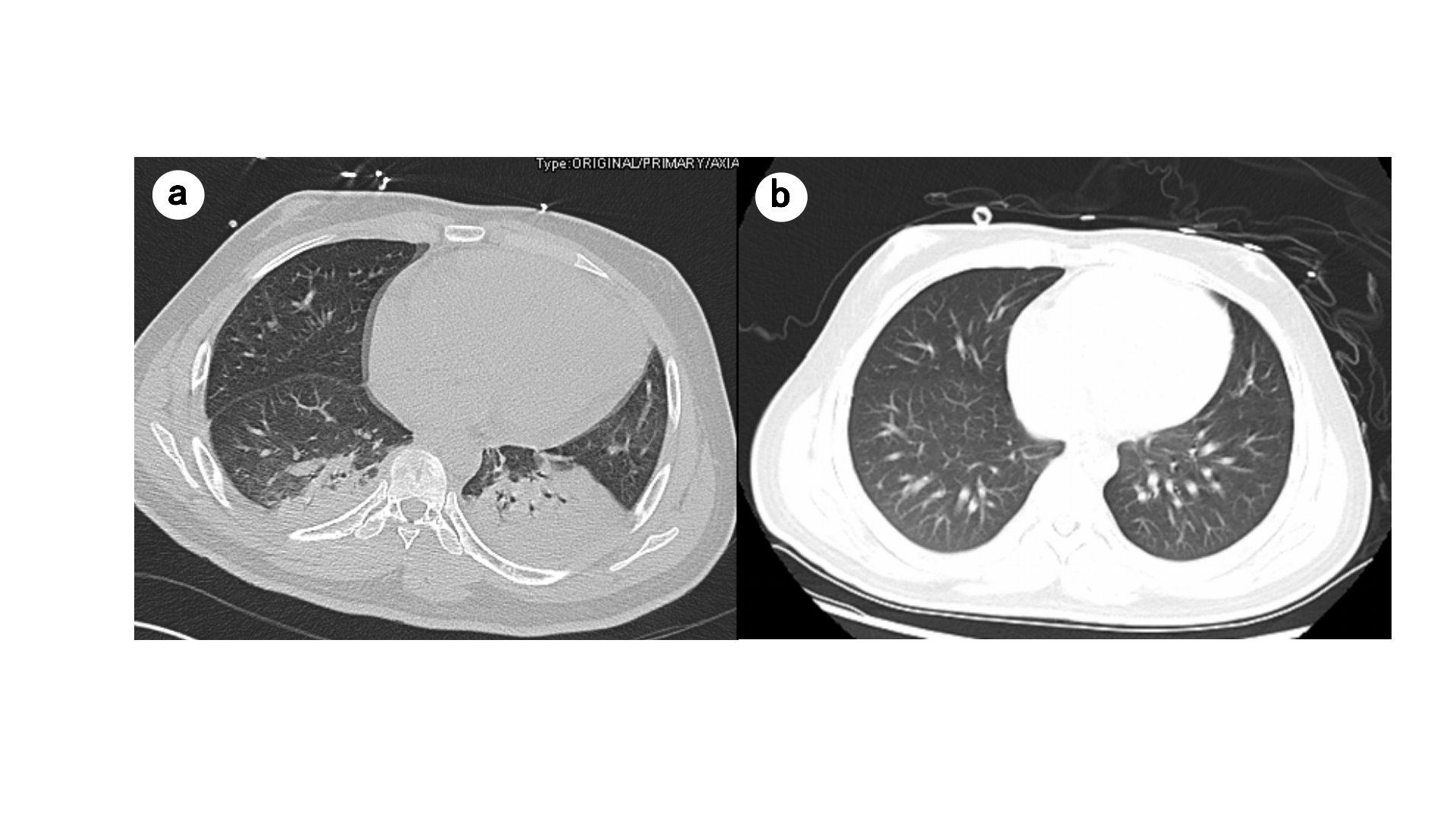

Supplement: Supplementary file 1 — Additional file 1: Figure S1. Chest CT on admission showed consolidation of lower lobes of both lungs (Panel a). Chest CT was normal after 20 days of antibacterial therapy (Panel b). [file 12879_2022_7529_MOESM1_ESM.tif]
