# Supplementary material for: Giant splenic cyst complicated by infection due to Salmonella enterica serovar Livingstone in a previously healthy adolescent male: a case report
Source: BMC Infect Dis. 2022 Jun 18;22:557. doi: 10.1186/s12879-022-07529-6 (PMC9206239; doi:10.1186/s12879-022-07529-6)
Supplement: Supplementary file 2 — Additional file 2: Table S1. Virulence factors of the strain. [file 12879_2022_7529_MOESM2_ESM.docx]

Table S1 Virulence factors of the strain.

| **VFclass** | **Virulence factors** | **Genes** | **VFclass** | **Virulence factors** | **Genes** | **VFclass** | **Virulence factors** | **Genes** |
| --- | --- | --- | --- | --- | --- | --- | --- | --- |
| Fimbrial adherence determinants | Agf/Csg | *csgA* | Fimbrial adherence determinants | Stb | *stbB* | Fimbrial adherence determinants | Sti | *stiA* |
|  |  | *csgB* |  |  | *stbC* |  |  | *stiB* |
|  |  | *csgC* |  |  | *stbD* |  |  | *stiC* |
|  |  | *csgD* |  |  | *stbE* |  |  | *stiH* |
|  |  | *csgE* |  | Stc | *stcB* |  | Stk | *stkA* |
|  |  | *csgF* |  |  | *stcC* |  |  | *stkB* |
|  |  | *csgG* |  | Std | *stdA* |  |  | *stkC* |
|  | Bcf | *bcfA* |  |  | *stdB* |  |  | *stkD* |
|  |  | *bcfB* |  |  | *stdC* |  |  | *stkE* |
|  |  | *bcfC* |  | Ste | *steA* |  |  | *stkF* |
|  |  | *bcfD* |  |  | *steB* |  |  | *stkG* |
|  |  | *bcfE* |  |  | *steC* | Macrophage inducible genes | Mig-14 | *mig-14* |
|  |  | *bcfF* |  |  | *steD* |  |  |  |
|  |  | *bcfG* |  |  | *steE* | Magnesium uptake | Mg2+ transport | *mgtB* |
|  | Fim | *fimA* |  |  | *steF* |  |  | *mgtC* |
|  |  | *fimC* |  | Stf | *stfA* | Nonfimbrial adherence determinants | MisL | *misL* |
|  |  | *fimD* |  |  | *stfC* |  | RatB | *ratB* |
|  |  | *fimF* |  |  | *stfD* |  | ShdA | *shdA* |
|  |  | *fimH* |  |  | *stfE* |  | SinH | *sinH* |
|  |  | *fimI* |  |  | *stfF* | Regulation | PhoPQ | *phoP* |
|  |  | *fimW* |  |  | *stfG* |  |  | *phoQ* |
|  |  | *fimY* |  | Sth | *sthA* | Adherence | Afimbrial adhesin AFA-I(Escherichia) | *afaA* |
|  |  | *fimZ* |  |  | *sthB* |  |  | *afaB* |
|  | Saf | *safB* |  |  | *sthC* |  |  | *afaC* |
|  |  | *safC* |  |  | *sthD* |  |  | / |
|  |  | *safD* |  |  | *sthE* |  |  | / |
| **VFclass** | **Virulence factors** | **Genes** | **VFclass** | **Virulence factors** | **Genes** | **VFclass** | **Virulence factors** | **Genes** |
| Secretion system | TTSS (SPI-1 encode) | *hilA* | Secretion system | TTSS (SPI-1 encode) | *spaP* | Secretion system | TTSS (SPI-2 encode) | *sseB* |
|  |  | *hilC* |  |  | *spaQ* |  |  | *sseC* |
|  |  | *hilD* |  |  | *spaR* |  |  | *sseD* |
|  |  | *iacP* |  |  | *spaS* |  |  | *sseE* |
|  |  | *iagB* |  |  | *sprB* |  |  | *ssrA* |
|  |  | *invA* |  | TTSS (SPI-2 encode) | *ssaC* |  |  | *ssrB* |
|  |  | *invB* |  |  | *ssaD* |  | TTSS effectors translocated via both systems | *slrP* |
|  |  | *invC* |  |  | *ssaE* |  | TTSS-1 translocated effectors | *sipA* |
|  |  | *invE* |  |  | *ssaG* |  |  | *sipB* |
|  |  | *invF* |  |  | *ssaH* |  |  | *sipC* |
|  |  | *invG* |  |  | *ssaI* |  |  | *sopA* |
|  |  | *invH* |  |  | *ssaJ* |  |  | *sopB/sigD* |
|  |  | *invI* |  |  | *ssaK* |  |  | *sopD* |
|  |  | *invJ* |  |  | *ssaL* |  |  | *sopE2* |
|  |  | *orgA* |  |  | *ssaM* |  |  | *sptP* |
|  |  | *orgB* |  |  | *ssaN* |  | TTSS-2 translocated effectors | *pipB2* |
|  |  | *orgC* |  |  | *ssaO* |  |  | *pipB* |
|  |  | *prgH* |  |  | *ssaP* |  |  | *sifA* |
|  |  | *prgI* |  |  | *ssaQ* |  |  | *sifB* |
|  |  | *prgJ* |  |  | *ssaR* |  |  | *sseF* |
|  |  | *prgK* |  |  | *ssaT* |  |  | *sseG* |
|  |  | *sicA* |  |  | *ssaU* |  |  | *sseJ* |
|  |  | *sicP* |  |  | *ssaV* |  |  | *sseK1* |
|  |  | *sipD* |  |  | *sscA* |  |  | *sseL* |
|  |  | *spaO* |  |  | *sscB* |  |  | *sspH2* |

TTSS, type III secretion systems.
